# Association between PM_2.5_ exposure and risk of Parkinson’s disease in individuals with chronic obstructive pulmonary disease in Taiwan: a nested case-control study

**DOI:** 10.4178/epih.e2023094

**Published:** 2023-10-17

**Authors:** Ci-Wen Luo, Yu-Hsiang Kuan, Wen-Ying Chen, Chun-Jung Chen, Frank Cheau-Feng Lin, Stella Chin-Shaw Tsai

**Affiliations:** 1 Department of Medical Research, Tungs’ Taichung MetroHarbor Hospital, Taichung, Taiwan; 2 Department of Pharmacology, Chung Shan Medical University School of Medicine, Taichung, Taiwan; 3 Department of Pharmacy, Chung Shan Medical University Hospital, Taichung, Taiwan; 4 Department of Veterinary Medicine, National Chung Hsing University, Taichung, Taiwan; 5 Department of Education and Research, Taichung Veterans General Hospital, Taichung, Taiwan; 6 Department of Thoracic Surgery, Chung Shan Medical University Hospital, Taichung, Taiwan; 7 School of Medicine, Chung Shan Medical University, Taichung, Taiwan; 8 Superintendent Office, Tungs’ Taichung MetroHarbor Hospital, Taichung, Taiwan; 9 Department of Post-Baccalaureate Medicine, National Chung Hsing University College of Medicine, Taichung, Taiwan

**Keywords:** Parkinson’s disease, Chronic obstructive pulmonary disease, Particulate matter, Nested case-control studies

## Abstract

**OBJECTIVES:**

This cohort study investigated the correlation between Parkinson’s disease (PD) risk and chronic obstructive pulmonary disease (COPD) risk under particulate matter with an aerodynamic diameter ≤2.5 μm (PM_2.5_) exposure.

**METHODS:**

Data from the National Health Research Institutes of Taiwan were used in this study. The Environmental Protection Administration of Taiwan established an air quality monitoring network for monitoring Taiwan’s general air quality. COPD was indicated by at least 3 outpatient records and 1 hospitalization for COPD. After the implementation of age, sex, and endpoint matching at a 1:4 ratio, 137 patients and 548 patients were included in the case group and control group, respectively. Based on the 2005 World Health Organization (WHO) standards, monthly air particle concentration data were classified into the following 4 groups in analyses of exposure–response relationships: normal level, and 1.0, 1.5, and 2.0 times the WHO level ([concentration ≥2]×25 μg/m^3^×number of exposure months).

**RESULTS:**

A multivariate logistic regression revealed that the 1.0 and 1.5 WHO level groups did not significantly differ from the normal level group, but the 2.0 WHO level did (odds ratio, 4.091; 95% confidence interval, 1.180 to 14.188; p=0.038).

**CONCLUSIONS:**

Elevated PM_2.5_ concentrations were significantly correlated with an increased risk of PD among patients with COPD. Furthermore, exposure to high PM_2.5_ levels can further increase the risk of PD.

## INTRODUCTION

Parkinson’s disease (PD) is a progressive neurodegenerative disorder with a typical presentation of slow progression and accumulating disability [1]. PD-related motor impairments, such as tremor, rigidity, slowed movement, and postural instability, can increase the likelihood of falls [2]. Cognitive impairments that affect executive functions, attention, and eventual memory involvement are predictors of poor clinical outcomes and an increased risk of developing dementia [3,4]. The incidence and prevalence of PD have been increasing rapidly worldwide. The global number of individuals with PD exceeded 6 million in 2015, and this number is projected to exceed 12 million by 2040 because of aging populations worldwide [5]. To date, PD has no disease-modifying treatment. Patients with PD not only require more physician consultations and emergency department visits, but also tend to have longer hospital stays relative to their same-age peers [6].

Chronic obstructive pulmonary disease (COPD) is a respiratory disease caused by exposure to inhaled particulate matter and a combination of multiple genetic, developmental, and social factors [7,8]. COPD is a key contributor to morbidity and mortality rates worldwide; in Taiwan, the mortality rate associated with COPD was 26.1 in every 100,000 people in 2014 [8]. For male aged > 55 years without COPD, the estimated risk of developing COPD within the next 40 years is 24% [9]. COPD is associated with numerous comorbidities, including coronary artery disease, heart failure, lung cancer, pulmonary artery disease, and malnutrition [10]. Other COPD-related comorbidities included systemic inflammatory metabolic diseases such as diabetes and hypertension and mental illnesses such as anxiety and depression [11]. A Taiwanese study also highlighted that compared with the general population, the risk of PD in patients with COPD was significantly higher [12].

Air pollution, particularly that caused by particulate matter with an aerodynamic diameter ≤ 2.5 μm (PM_2.5_), is a risk factor associated with COPD. Short-term exposure to air pollution exacerbates COPD [11], and older adults are more sensitive to adverse reactions, with the estimated risk being higher in European countries than in Asian countries. Various other confounding factors also contribute to the inconsistent results [13,14]. Exposure to annual average PM_2.5_ in the United States is significantly associated with an increased risk of first hospitalization for PD, Alzheimer’s disease, and related dementia [15]. C57BL/6J mice have been used as an in vivo model to show that PM_2.5_ exposure exacerbates behavioral abnormalities in individuals with PD by increasing oxidative stress, reducing autophagy and mitophagy, and inducing mitochondrial-mediated neuronal apoptosis [16].

Studies have suggested a relationship between greater PM_2.5_ exposure and higher COPD and PD risk and confirmed the higher risk of PD development in individuals with COPD. Our study aimed to investigate whether PD risk was correlated with exposure to PM_2.5_ in COPD patients.

## MATERIALS AND METHODS

### Data sources

This cohort study analyzed data obtained from the National Health Research Institutes of Taiwan, specifically the National Health Insurance Research Database (NHIRD). Since 1995, Taiwan’s National Health Insurance program has provided universal and publicly funded health coverage to the country’s population, which is currently approximately 23 million. This program is mandatory and covers more than 98% of Taiwan’s population [17]. The NHIRD includes records of both hospital admissions and outpatient visits, and clinicians apply the International Classification of Diseases, 9th revision, Clinical Modification (ICD-9-CM), to assign codes to the data. The database has been validated by numerous studies. Because of the encryption and de-identification processes implemented by the NHIRD, patient privacy is protected.

### Collection of particulate matter with an aerodynamic diameter ≤2.5 μm concentration data

The Environmental Protection Administration of Taiwan established an air quality monitoring network (AMQN) to monitor the general air quality in Taiwan. In this monitoring network, the PM_2.5_ concentration in the atmosphere is measured using a tapered element oscillating microbalance (R&P 1400; Rupprecht & Patashnick Co., New York, NY, USA) and measurements are recorded once per hour. The time interval for follow-up was measured in months, and the present study used monthly average cumulative exposure to compare the PM_2.5_ exposure levels of the studied patients. A patient’s exposure was determined using the exposure measurement taken at the monitoring station where the patient was located. If a patient’s location did not have a monitoring station, the patient’s exposure was determined using the measurement taken at the nearest monitoring station or the average of the 2 nearest monitoring stations. The observed air particle exposure levels that the studied patients were exposed to were determined on the basis of the monthly average cumulative exposure levels from the year 2008 to the endpoint. The hourly cumulative exposure level multiplied by 24 was used as the basis for daily exposure. If more than 8 hours of data were missing, the patient was excluded. The monthly average exposure level for a given month was calculated by multiplying the average daily exposure by the number of days in that month. If more than 10 days of data were missing, the patient was excluded. The monthly average air particle concentration at a given city was estimated and regarded as the population exposure of the city. We referenced studies that used quartiles to establish a basis for grouping exposure concentrations [18]. To analyze the exposure–response relationship, the monthly concentration of air particles was classified into the following 4 categories by applying the 2005 World Health Organization (WHO) standards: normal level (≤25 μg/m^3^ ×number of exposure months), 1.0 times the WHO level ([1≤ concentration< 1.5]× [25 μg/m^3^ × number of exposure months]), 1.5 times the WHO level (1.5≤ concentration< 2]× [25 μg/m^3^ × number of exposure months]), and 2.0 times the WHO level ([concentration≥ 2]× 25 μg/m^3^ × number of exposure months).

### Study population

**Figure 1.**
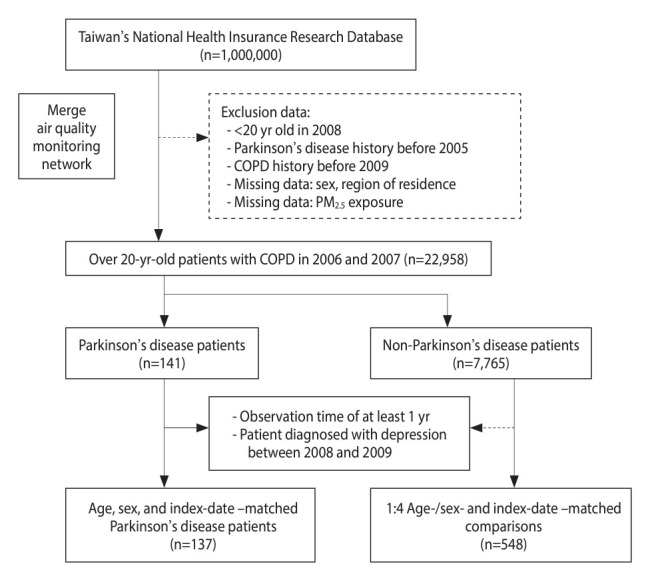
Flow diagram for study participants. COPD, chronic obstructive pulmonary disease. PM_2.5_, particulate matter with an aerodynamic diameter ≤2.5 μm.

From the NHIRD and the AQMN database, the present study obtained data on the PM_2.5_ exposure and population characteristics of patients with COPD for the period from 2006 to 2013. Individuals aged ≥ 20 years with no history of COPD (ICD-9 codes: 491.x, 492.x, and 496.x) prior to 2005 were enrolled between 2006 and 2007 [19]. COPD was indicated by at least 3 outpatient records and 1 hospitalization record for COPD. In total, 8,160 patients were diagnosed with COPD, and those with missing values were excluded (Figure 1). Among these patients, 141 were diagnosed with PD (ICD-9 code: 332.0), and 7,765 were undiagnosed with PD [20]. In the present nested case-control study, patients who were diagnosed with PD within 365 days between 2008 and 2009 were excluded from the case group, and their counterparts were excluded from the control group. Patients with missing PM_2.5_ data were also excluded from both groups. We matched the case group with a control group that was 4 times larger. After the implementation of 1:4 matching for age, sex, and index date, 137 patients and 548 patients were included in the case group and control group, respectively.

The basic participant characteristics examined in the present study were sex, age, low income, and urbanization level. In Taiwan, households with a monthly income of < 20,000 New Taiwan dollars [21] are exempt from paying income tax. The 359 registered communities in Taiwan are divided into 7 urbanization levels based on their composite scores for population density, education level ratio, proportion of older adults aged ≥ 65 years, proportion of the community engaged in agricultural activities, and the number of individuals with COPD per 100,000 people [22]. The 7 urbanization levels are highly urbanized, moderate urbanization, emerging town, general town, aging towns, agrarian towns, and remote township. The comorbidities associated with COPD include diabetes, hypertension, ischemic stroke, fracture, depression, cancer, and Alzheimer’s disease [23].

### Statistical analysis

We performed various statistical tests to analyze the data. The Shapiro–Wilk test was applied to identify whether the data distribution was non-normal (p< 0.05), and the Wilcoxon rank-sum test was conducted to assess differences in continuous variables between the case and control groups. The chi-square test was performed to analyze differences in categorical variables between the case and control groups. To calculate the odds ratios (ORs) and 95% confidence intervals (CIs), we used multivariate logistic regression models. These models were adjusted for potential risk factors such as PD-related comorbidities, age, sex, low income, and urbanization level. Specifically, we employed multivariate logistic regression to estimate the OR and 95% CI for PD risk in patients with COPD who were exposed to PM_2.5_. We performed all statistical analyses using the SAS version 9.4 (SAS Institute Inc., Cary, NC, USA), and p< 0.05 indicated statistical significance.

### Ethics statement

The present study was approved by the Institutional Review Board of Tungs’ Taichung MetroHarbor Hospital (IRB No. 111,070).

## RESULTS

### Patient characteristics

Table 1 presents the basic characteristics of PM_2.5_ exposure among patients in the case group (PD) and comparison group (non-PD). The patients with PD who were diagnosed with COPD between 2006 and 2007 in the NHIRD were followed up from 2008 to 2013 after matching was performed for age (±5 years), sex, and index date (±180 days). No significant difference between the case and control groups was detected for sex, age, and low income. The 2 groups also did not differ significantly with respect to urbanization level; however, a high proportion of both groups lived in highly urbanized cities, with 28.5% of the patients with PD and 26.6% of those without PD living in those areas. For comorbidities, significant between-group differences were detected for diabetes and anemia, with a significantly higher proportion of the patients with PD (diabetes, 28.4%; anemia, 16.7%) experiencing these conditions relative to the patients; thus, these were identified as risk factors for PD (diabetes, 21.6%; anemia, 11.5%).

### Particulate matter with an aerodynamic diameter ≤2.5 μm exposure as risk factor for Parkinson’s disease

Table 2 shows the distribution of WHO PM_2.5_ levels among the case and control groups. The mean follow-up period was 42.42±16.79 months for the patients with PD and 42.50±16.60 months for the patients without PD. At normal levels, the median PM_2.5_ exposure was 1,023.57 μg/m³ (mean±standard deviation [SD], 940.96±321.74) for 13 patients with PD and 834.12 μg/m³ (mean±SD, 778.54±289.57) for 54 patients without PD. At the WHO 1.0 level, the median PM_2.5_ exposure was 1,354.78 μg/m³ (mean±SD, 1,315.02 ±590.59) for 80 patients with PD and 1,279.43 μg/m³ (mean±SD, 1,310.19±586.53) for 356 patients without PD. At the WHO 1.5 level, the median PM_2.5_ exposure was 1,426.56 μg/m³ (mean ±SD, 1,582.35 ±702.32) for 37 patients with PD and 1,746.72 μg/m³ (mean±SD, 1,723.57±683.53) for 130 patients without PD. At the WHO 2.0 level, the median PM_2.5_ exposure was 2,276.40 μg/m³ (mean±SD, 2,197.08±179.38) for 7 patients with PD and 2,226.65 μg/m³ (mean±SD, 2,220.99±129.18) for 8 patients without PD. The chi-square test revealed a significant difference in PM_2.5_ exposure levels between the PD and control groups.

### Determination of the odds ratio of particulate matter with an aerodynamic diameter ≤2.5 μm exposure level as risk factor for chronic obstructive pulmonary disease through logistic regression

Table 3 presents the confounding variables for the risk of developing PD in patients with COPD who were exposed to PM_2.5_. The multivariate logistic regression revealed that the WHO 1.0 and 1.5 level groups did not significantly differ from the normal level group, but the WHO 2.0 level group did (OR, 4.091; 95% CI, 1.180 to 14.188; p= 0.026). No significant difference was observed for sex, age, and low income. PD risk did not significantly differ between areas with and without high urbanization; however, a higher trend was observed for aging towns (OR, 2.333; 95% CI, 0.868 to 6.268). Only the comorbidities of anemia (OR, 1.606; 95% CI, 0.998 to 2.584) and diabetes (OR, 1.458; 95% CI, 0.966 to 2.201) were significantly associated with PD risk.

## DISCUSSION

The body of evidence on the link between air pollution and neurological disorders is growing, particularly with respect to the link between ozone and particulate matter exposure and brain diseases [24]. This includes the activation of microglial cells and other inflammatory markers, elevated levels of midbrain α-synuclein protein, and the loss of dopamine neurons in the substantia nigra [25-27]. Progressive damage occurs in various regions of the brain, and such damage is accompanied by behavioral changes and small glial cell activation, changes in the morphology of the substantia nigra and striatum cells, and the loss of neurons [28,29]; these changes are similar to those observed in the brains of patients with PD [30].

PM_2.5_ is a crucial contributor to the risk of not only PD, but also cardiovascular disease and other related chronic diseases such as COPD [16]. Studies have reported that exposure to PM_2.5_ induces lung inflammation in mice and exacerbates cigarette-smoke-induced inflammation [31]. The levels of interleukin 6 and 8 were reported to be upregulated in PM_2.5_-exposed cells in a dose-dependent manner, impairing the transition of AT2 cells to AT1 cells in a mouse model [31]. Various epidemiological studies have demonstrated that exposure to particulate matter pollution, including PM_2.5_, is associated with numerous chronic diseases in both developed and developing countries [18]. In particular, PM_2.5_ can pass through the blood–lung barrier and blood–brain barrier, thereby increasing the incidence of respiratory, cardiovascular, and neurodegenerative diseases [19]. The neuroinflammatory process can be attributed to the ability of PM_2.5_ particles to reach the nasal epithelium through respiration and to transfer to the brain through the olfactory bulb [20]. Our research findings indicated a correlation between an elevated PM_2.5_ concentration and an increased risk of PD among patients with COPD. Furthermore, exposure to high levels of PM_2.5_ can further elevate the risk of PD.

PD is the second most common age-related neurodegenerative disorder, affecting approximately 3% and 5% of the populations aged > 65 years and > 85 years, respectively [21]. Males have a 2-fold greater risk of developing PD relative to females, but female exhibit a higher mortality rate; furthermore, COPD is more hazardous for male than for female [23,32]. In a recent meta-analysis, age-related increases in PD incidence were observed in both sexes, but males in the 60-year-old to 70-year-old age group exhibited a faster increase relative to individuals in the other age groups [24]. Our results indicated that relative to females, males constituted a larger proportion of the study population, tended to be older, and exhibited greater PD risk if they were living in an aged township, even though this trend was non-significant. However, because of the limitations related to our research methodology, we could not clarify the correlation between PD and COPD risk factors. Compared with winter births, spring births were reported to have a 30% higher risk of PD [25]. The amount of early-life sunlight exposure and subsequent vitamin D3 levels of individuals may influence their risk of developing PD [26]. In a United Kingdom study, non-motor symptoms in patients with PD exhibited seasonal fluctuations, particularly symptoms related to cardiovascular, sensory, hallucinatory, and olfactory functions, and such symptoms worsened considerably in the winter [27]. Based on the possible seasonal and sunlight-related PD trends reported by one study, we treated birth season and diagnosis season as confounding factors and made the necessary adjustments. Although differences in the number of PD cases were observed across birth and diagnosis seasons, they were non-significant.

Numerous studies have reported that diabetes mellitus increases the risk of PD. Insulin plays a crucial role in protecting neurons during neuron development by binding to its receptors [28]. In PD cell models, insulin reduces the BAX/BAL2 ratio by activating the phosphatidylinositol-3-kinase/protein kinase B/glycogen synthase kinase 3 pathway [29]. These pathophysiological links include neuroinflammation, mitochondrial dysfunction, oxidative stress, and protein misfolding processes [30]. Insulin resistance and hyperglycemia are the underlying causes of most comorbidities related to diabetes mellitus, and they also have negative effects on PD. Oxidative stress can lead to anemia because it affects lipid peroxidation and DNA damage [33], contributing to the development of anemia under these pathological conditions [34]. The disruption of iron homeostasis can lead to various neurodegenerative diseases, including PD. A preclinical study reported that mice with gene knockout of iron-regulating proteins that regulate cell iron homeostasis developed progressive neurodegeneration [35]. Males with anemia were reported to have a higher incidence of PD; however, this trend was not observed in females, which may be related to gynecological diseases. Our results indicated that after adjustments were made, no significant relationship was detected between diabetes and anemia, which may reflect the effects of hormones [36]; however, the patients with diabetes and anemia had a higher risk for PD than their counterparts.

The present study has several limitations. First, no laboratory data were collected. Data pertaining to biochemical markers such as eosinophil count and glycosylated hemoglobin levels could not be collected from the patients. However, adjustments were made for comorbidity-related confounding factors to minimize the influence of this limitation on our results. Second, the patients’ PM_2.5_ exposure data could be inaccurate to some extent. PM_2.5_ exposure was determined on the basis of the patients’ residential areas; however, some patients spent time in other areas during the study period, which would have affected their exposure to PM_2.5_. However, for long-term exposure, the patients’ main activity areas were likely to be close to their place of residence; thus, this limitation did not have a substantial effect on our results. Third, the details of the patients’ lifestyle habits (such as smoking and drinking) are not recorded in the NHIRD, and lifestyle-related factors could have influenced the results. However, we adjusted for socioeconomic status, comorbidities, and urbanization level, and these factors did not lead to significant deviations from our results.

The present study identified a concentration-dependent relationship between PM_2.5_ exposure and the risk of PD in patients with COPD, with a higher concentration of PM_2.5_ leading to a greater risk of PD. These results highlight the necessity of considering neurodegenerative diseases in the context of environmental pollution and respiratory symptoms.

## Figures and Tables

**Table 1.** Demographic characteristics of COPD patients with and without Parkinson’s disease

Characteristics		Non-PD (n=548)	PD (n=137)	p-value
Sex				
	Female	228 (41.6)	57 (41.6)	>0.999
	Male	320 (58.4)	80 (58.4)	
Age				
	Mean±SD	71.99±10.00	72.41±10.45	0.143
Low income				
	Yes	366 (46.4)	103 (52.3)	0.111
	No	422 (53.5)	94 (47.7)	
Urbanization level				
	Highly urbanized	210 (26.6)	34 (24.8)	0.142
	Moderate urbanization	228 (28.9)	39 (28.5)	
	Emerging town	169 (21.4)	20 (14.6)	
	General town	106 (13.4)	20 (14.6)	
	Aging towns	10 (1.3)	9 (6.6)	
	Agrarian towns	40 (5.1)	10 (7.3)	
	Remote township	25 (3.2)	5 (3.6)	
Season				
	Born in spring	122 (15.5)	36 (18.3)	0.318
	Diagnosed in winter	119 (15.1)	33 (16.7)	0.550
Comorbidities				
	Diabetes	170 (21.6)	56 (28.4)	0.028
	Hypertension	389 (49.4)	105 (53.3)	0.187
	Ischemic stroke	22 (2.8)	8 (4.1)	0.350
	Fracture	126 (16.0)	30 (15.2)	0.785
	Depression	14 (1.8)	6 (3.0)	0.256
	Anemia	91 (11.5)	33 (16.7)	0.042
	Sleep disturbances	211 (26.8)	65 (33.0)	0.893
	Malignant disease	83 (10.5)	20 (10.1)	0.873
	Alzheimer’s disease	11 (1.4)	3 (1.5)	0.318

Values are presented as number (%). COPD, chronic obstructive pulmonary disease; PD, Parkison’s disease; SD, standard deviation.

**Table 2.** Particulate matter 2.5 levels in COPD patients with and without Parkinson’s disease

PM_2.5_ exposure level		n (%)	Distribution of PM_2.5_ (μg/m^3^)				p-value^1^
			Mean±SD	Q1	Median	Q3	
Parkinson’s disease (follow-up: 42.42±16.79 mo)							0.048
	Normal level	13 (9.5)	940.96±321.74	663.57	1,023.57	1,138.76	
	1.0 WHO level	80 (58.4)	1,315.02±590.59	807.72	1,354.78	1,735.38	
	1.5 WHO level	37 (27.0)	1,582.35±702.32	1,073.75	1,426.56	2,066.99	
	2.0 WHO level	7 (5.1)	2,197.08±179.38	2,007.95	2,276.40	2,375.88	
Non-Parkinson’s disease (follow-up: 42.50±16.60 mo)							
	Normal level	54 (9.8)	778.54± 289.57	578.25	834.12	967.49	
	1.0 WHO level	356 (65.0)	1,310.19±586.53	815.17	1,279.43	1,736.23	
	1.5 WHO level	130 (23.7)	1,723.57±683.53	1,147.51	1,746.72	2,291.85	
	2.0 WHO level	8 (1.5)	2,220.99±129.18	2,148.92	2,226.65	2,326.14	

COPD, chronic obstructive pulmonary disease; PM_2.5_, particulate matter with an aerodynamic diameter ≤2.5 μm; SD, standard deviation; WHO, World Health Organization.

1 Chi-square test.

**Table 3.** Logistic regression of PM_2.5_ levels and Parkinson’s disease^1^

Variables		Parkinson’s disease	p-value
PM_2.5_ level (reference: Q1 level)			
	1.0 WHO level	0.947 (0.474, 1.892)	0.877
	1.5 WHO level	1.200 (0.570, 2.528)	0.631
	2.0 WHO level	4.091 (1.180, 14.188)	0.026
Sex (reference: female)			
	Male	1.086 (0.720, 1.638)	0.693
Age		0.997 (0.977, 1.019)	0.812
Low-income (reference: no)			
	Yes	1.383 (0.871, 2.196)	0.170
Urbanization level (reference: highly urbanized)			
	Moderate urbanization	0.759 (0.434, 1.327)	0.334
	Emerging town	0.571 (0.301, 1.083)	0.086
	General town	0.687 (0.348, 1.355)	0.279
	Aging towns	2.333 (0.868, 6.268)	0.093
	Agrarian towns	0.957 (0.400, 2.293)	0.922
	Remote township	0.674 (0.229, 1.986)	0.474
Season (reference: no seasonal variation)			
	Born in the spring	1.354 (0.862, 2.128)	0.188
	Diagnosed in winter	1.212 (0.764, 1.922)	0.414
Comorbidities (reference: without)			
	Diabetes	1.458 (0.966, 2.201)	0.072
	Hypertension	1.240 (0.763, 2.015)	0.386
	Ischemic stroke	1.438 (0.604, 3.423)	0.412
	Fracture	0.910 (0.561, 1.477)	0.703
	Depression	1.676 (0.603, 4.661)	0.322
	Anemia	1.606 (0.998, 2.584)	0.051
	Sleep disturbances	1.221 (0.813, 1.834)	0.336
	Malignant disease	0.988 (0.568, 1.719)	0.965
	Alzheimer’s disease	0.873 (0.227, 3.352)	0.843

Values are presented as adjusted odds ratio (95% confidence interval). PM_2.5_, particulate matter with an aerodynamic diameter ≤2.5 μm; WHO, World Health Organization.

1 Adjustment for sex, age, low-income, urbanization level, comorbidities.
